# Association between COVID-19 Primary Vaccination and Severe Disease Caused by SARS-CoV-2 Delta Variant among Hospitalized Patients: A Belgian Retrospective Cohort Study

**DOI:** 10.3390/vaccines11010014

**Published:** 2022-12-21

**Authors:** Queeny Robalo, Laurane De Mot, Mathil Vandromme, Nina Van Goethem, Andrea Gabrio, Pui Yan Jenny Chung, Marjan Meurisse, Belgian Collaborative Group on COVID-19 Hospital Surveillance, Lucy Catteau, Carel Thijs, Koen Blot

**Affiliations:** 1 Scientific Directorate of Epidemiology and Public Health, Sciensano, 1050 Brussels, Belgium; 2 Natuurpunt Studie vzw, 2800 Mechelen, Belgium; 3 Department of Methodology and Statistics, Care and Public Health Research Institute (CAPHRI), Faculty of Health, Medicine and Life Sciences (FHML), Maastricht University, 6229 ER Maastricht, The Netherlands; 4 Maastricht University Medical Centre+, Department of Epidemiology, Care and Public Health Research Institute (CAPHRI), Faculty of Health, Medicine and Life Sciences (FHML), Maastricht University, 6229 ER Maastricht, The Netherlands

**Keywords:** SARS-CoV-2, COVID-19, Delta, hospitalized, vaccine, brand

## Abstract

We aimed to investigate vaccine effectiveness against progression to severe COVID-19 (acute respiratory distress syndrome (ARDS), intensive care unit (ICU) admission and/or death) and in-hospital death in a cohort of hospitalized COVID-19 patients. Mixed effects logistic regression analyses were performed to estimate the association between receiving a primary COVID-19 vaccination schedule and severe outcomes after adjusting for patient, hospital, and vaccination characteristics. Additionally, the effects of the vaccine brands including mRNA vaccines mRNA-1273 and BNT162b2, and adenovirus-vector vaccines ChAdOx1 (AZ) and Ad26.COV2.S (J&J) were compared to each other. This retrospective, multicenter cohort study included 2493 COVID-19 patients hospitalized across 73 acute care hospitals in Belgium during the time period 15 August 2021–14 November 2021 when the Delta variant (B1.617.2) was predominant. Hospitalized COVID-19 patients that received a primary vaccination schedule had lower odds of progressing to severe disease (OR (95% CI); 0.48 (0.38; 0.60)) and in-hospital death (OR (95% CI); 0.49 (0.36; 0.65)) than unvaccinated patients. Among the vaccinated patients older than 75 years, mRNA vaccines and AZ seemed to confer similar protection, while one dose of J&J showed lower protection in this age category. In conclusion, a primary vaccination schedule protects against worsening of COVID-19 to severe outcomes among hospitalized patients.

## 1. Introduction

Vaccines were developed in record time in response to the detection of severe acute respiratory syndrome coronavirus-2 (SARS-CoV-2) to decrease viral circulation, prevent symptomatic disease, and protect against severe COVID-19 outcomes [1]. In Belgium, as of November 2022, 4,619,721 confirmed cases, 140,382 hospitalizations, and 32,959 deaths due to COVID-19 have been reported [2]. The clinical spectrum after SARS-CoV-2 infection ranges from asymptomatic infection to acute respiratory distress syndrome (ARDS) and death. In general, the clinical course in hospitalized COVID-19 patients can roughly be separated into four main phases: an early viral phase, a phase defined by cytokine release syndrome, a phase during which coagulopathy arises, and finally, a phase with several possible outcomes such as multi-organ failure including acute respiratory distress syndrome (ARDS), death, full recovery, or slow, suboptimal recovery with a post-COVID-19 syndrome [3]. Apart from preventing transmission, COVID-19 vaccines were designed to protect against severe outcomes. The four COVID-19 vaccines initially used in Belgium include vaccines based on either a replication-deficient adenoviral vector carrying the spike SARS-CoV-2 protein or on mRNA technology with delivery of the receptor binding domain sequence (part of the spike protein). The Spikevax/mRNA-1273 vaccine designed by Moderna (MOD) and Comirnaty/BNT162b2 by Pfizer/BioNTech (COM) make use of mRNA technology [4] and are referred to as mRNA vaccines throughout this text. Vaxzevria/AZD1222, developed by AstraZeneca/Oxford (AZ), and Janssen COVID-19 vaccine/Ad26.COV2.S by Johnson & Johnson (J&J) consist of a non-replicating adenoviral vector. The mRNA vaccines and AZ require a two-dose vaccination schedule, while the J&J vaccine was marketed as a one-dose vaccine.

**Figure 1:**
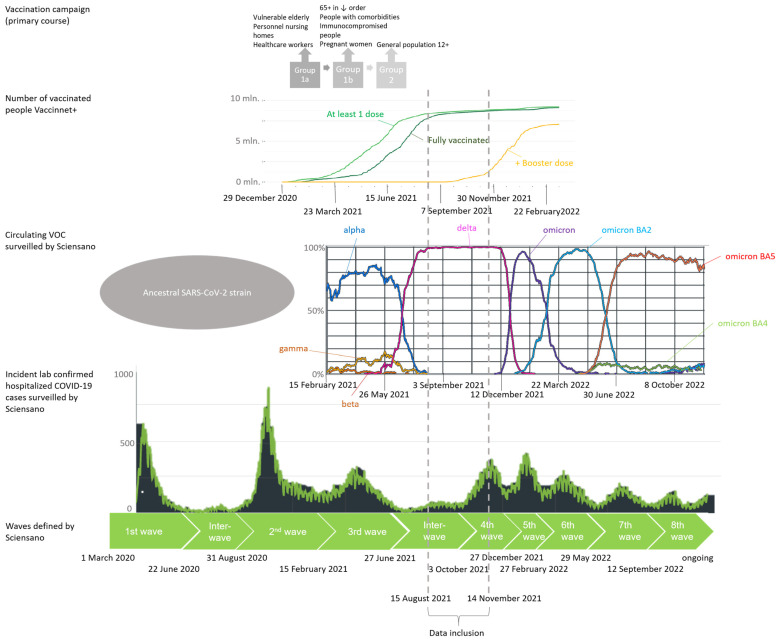
COVID-19 epidemiologic situation in Belgium: timeline. Schematic view of different waves in the COVID-19 epidemic in Belgium determined by circulating variants of concern (VOCs), implemented measures of restriction, number of diagnosed cases, number of hospitalizations, number of occupied beds in the hospitals and intensive care units (ICU) and speed of increase in cases [8,31]. The original Belgian vaccination campaign that started in January 2021 and stagnation of second dose delivery in August 2021 is included to indicate which high-risk groups received priority in exposure to the vaccines [5] (p. 39). Vaccine uptake: light green line: at least 1 dose; darker green line: fully vaccinated; yellow: additional booster dose. VOC: dark blue line: Alpha strain; orange line: Beta strain; yellow line: Gamma strain; pink line: Delta strain; purple line: Omicron strain; teal blue line: Omicron BA2 strain; light green line: Omicron BA4; red line: Omicron BA5.

The Belgian vaccination campaign was launched in January 2021 and prioritized high-risk groups in the following order: nursing home residents and personnel, collective care institutions, healthcare workers, persons aged ≥65 years, persons between 45–65 years with comorbidities, and persons employed in jobs critical to society and economy (Figure 1) [5] (p. 39). The vaccination campaign was then extended to the general population aged ≥18 years. As of October 2022, a total of 9,103,043 people have been vaccinated with the primary vaccination course in Belgium, corresponding to a vaccination coverage of 78.6% [6].

Despite high vaccine coverage [7], the variant of concern (VOC) Delta (B.1.617.2), first detected in October 2020 in the Maharashtra state of India, caused a third wave of COVID-19 infections in Belgium between 15 February 2021 and 27 June 2021 [8]. This Delta wave was partially characterized by a surge of breakthrough infections among vaccinated people. While vaccines were developed against the ancestral, wild-type strain discovered in Wuhan, four major amino acid changes in the spike protein of Delta enabled this VOC to escape immunity and decrease vaccine effectiveness [9]. Still, even with the Delta VOC, vaccinated persons with breakthrough infections often had asymptomatic or milder disease compared to unvaccinated persons [7,10,11]. Several studies have also reported a faster decline in viral RNA load, when adjusting for time since vaccination [12], which indicates that the vaccines are beneficial for the decrease of viral transmission [10,13,14,15,16]. In addition, the vaccines have proven to be effective against severe disease and death, despite the surge in hospitalizations associated with the emergence of the Delta variant [4,7,10,17,18,19,20,21,22,23,24]. Harder et al. estimated that, based on 17 observational studies, the pooled vaccine effectiveness against the Delta variant was 63.1% (40.9–76.9) against asymptomatic infection, 75.7% (69.3–80.8) against symptomatic COVID-19 and 90.9% (84.5–94.7) against hospitalization [7]. 

Although many vaccine effectiveness studies and descriptive studies have been conducted, the present study was intended to provide more real-world evidence on brand comparison information that is currently lacking in the context of COVID-19 progression to severe outcomes. We investigated this in the Belgian population with data of high quality through linkage of extensive person-level databases, which made correction for confounding data and restriction to one VOC and specific vaccine brands possible. The first goal of this study was to investigate the effects of the COVID-19 vaccines against progression to severe COVID-19 (defined as presence of ARDS, ICU admission/transfer and/or death), and to all-cause in-hospital death in a cohort of patients hospitalized due to a SARS-CoV-2 Delta infection, using the nationwide Belgian surveillance system. Secondarily, we aimed to assess the importance of vaccine type (mRNA vaccines versus adenovirus-vector vaccines) in these outcomes.

## 2. Materials and Methods

### 2.1. Study Design and Data Sources

This multicenter, hospital-based, retrospective cohort study was part of the LINK-VACC project initiated by Sciensano in Belgium. The LINK-VACC project links several registries such as: (1) the Clinical Hospital Survey database (CHS) [25], which contains patient-level information from admission and discharge forms of approximately 50% of the COVID-19 patients admitted across 103 Belgian hospitals, (2) the Surge Capacity Survey database (SCS) [25], containing exhaustive hospital-level information on COVID-19 prevalence, incidence, and mortality deducted from aggregated numbers of admissions, discharges, and bed occupancy, (3) Vaccinnet+, containing information on COVID-19 vaccination statuses of all vaccinated Belgian citizens, and (4) a database with results of all recorded reverse transcriptase-polymerase chain reaction (RT-PCR) and rapid antigen tests (RAT) in Belgium for detection of SARS-CoV-2. Further information about the linking of these databases within the context of the established LINK-VACC framework is provided by Van Goethem et al. [26].

### 2.2. Study Population

The study population consisted of COVID-19 patients admitted to a Belgian hospital between 15 August 2021 and 14 November 2021 and for whom an admission was reported in the Clinical Hospital Survey (CHS). The CHS allows surveillance of 103 acute care hospitals across Belgium, but for the period chosen, as well as after applying in- and exclusion criteria for the cohort in this study, data were obtained from 73 hospitals. The starting date for data inclusion corresponds to a plateau in the vaccine coverage among the Belgian population, with approximately 85% of all Belgian citizens ≥ 18 years having received a complete primary vaccination course [5] (p. 39) (Figure 1: Vaccination campaign). The end date for data inclusion was chosen to restrict on infections with the Delta variant since after that date the Omicron variant was increasingly detected in Belgium [27,28,29,30,31]. Within this period, 99–100% of circulating strains were Delta variants according to the representative genomic baseline surveillance [27,28,29,30,31].

The following inclusion and exclusion criteria were applied to select the cohort that was used in the final complete case analyses. We included patients hospitalized due to a confirmed COVID-19 infection (RT-PCR or RAT) with complete information on admission, discharge, and vaccination status available. In other words, patients that were admitted due to a reason other than COVID-19 (including when having a positive diagnosis after systematic screening for SARS-CoV-2) were excluded. Among the excluded group were patients with asymptomatic infections and mild COVID-19 and patients that acquired a nosocomial SARS-CoV-2 infection with the date of diagnosis more than 1 day after the date of hospital admission. The European Center for Disease Prevention and Control (ECDC) classifies SARS-CoV-2 infections as definite healthcare-associated, probable healthcare-associated, indeterminate COVID-19 cases, community-acquired, and nursing home-acquired infections [32]. In compliance with these definitions, the first three types of (nosocomial) infections were excluded to keep the focus on community-acquired, nursing-home acquired, and infections of unknown origin in healthcare workers. Additionally, COVID-19 diagnoses only based on chest CT scans were considered insufficient, so patients diagnosed in this way were excluded. Receiving a vaccine other than the vaccines of interest for this study (mRNA vaccines, AZ, or J&J) or a mixed vaccine brand schedule was an exclusion criterion. Partially vaccinated patients (exposure to only one dose in case not with J&J and/or confirmed SARS-CoV-2 infection at hospital admission within 14 days after finishing the primary vaccination schedule) and boosted vaccinated patients (more than two vaccine doses in case not with J&J or more than one dose in case of J&J) were also excluded. Finally, children and young adults < 18 years old, pregnant, or post-partum (<6 weeks) women, patients that had experienced documented previous SARS-CoV-2 infections were excluded. Patients admitted to a military or psychiatric hospital, an unverified hospital, or hospital without ICU as well as hospital transferred, and readmitted patients were excluded.

### 2.3. Operationalization of Variables

#### 2.3.1. Outcomes

Two outcomes were assessed in this cohort: (1) severe COVID-19 defined by ICU admission, diagnosis of ARDS and/or all-cause in-hospital death, and (2) all-cause in-hospital death. Diagnosis of ARDS was made based on the definition of Berlin [33]. This definition is also used by the WHO for hospitalized, severe COVID-19 disease with ARDS [34]. Our outcome, severe COVID-19, corresponds to levels 8–10 of the minimal outcome set by the WHO with level 10 equal to death [34]. In this study, ICU admission, diagnosis of ARDS, and in-hospital death were operationalized as dichotomous (yes/no) variables. Patients with ‘yes’ for one or more of these three variables were classified as severe COVID-19 cases.

#### 2.3.2. Exposure

Exposure was defined in this study as vaccination with two doses of mRNA vaccines or AZ, or one dose of J&J, at least 14 days before the date of infection with SARS-CoV-2. Vaccination status was operationalized as a dichotomous variable (primary vaccination course/unvaccinated) in the primary analyses. In the secondary analyses on the DBC subcohort, vaccine brands (mRNA vaccines/AZ/J&J) were compared to each other with viral vector vaccine J&J as reference category. A Delta breakthrough case (DBC) was defined as an individual that received a primary vaccination schedule, yet developed a breakthrough infection resulting in COVID-19 that was severe enough to require hospitalization.

#### 2.3.3. Covariates

The following covariates were included in the primary and secondary analyses: age, sex, comorbidities (cardiovascular disease, arterial hypertension, diabetes, chronic renal disease, chronic liver disease, chronic neurologic or neuromuscular disease, cognitive impairment, immunocompromised disease (including HIV and chronic corticosteroid use), chronic lung disease, solid cancer, hematological cancer, solid organ transplantation, and obesity), nursing home residency, and mean ICU occupancy rate. Time since vaccination was added to the models in the secondary analyses.

Age (in years), and the previously listed comorbidities (presence/absence), were considered as potential confounders and were therefore included in all models. These patient characteristics are correlated with the probability of being vaccinated, the timing (priority groups and vaccine eligibility reasons), and with the vaccine type (mRNA vaccines were distributed the most and throughout the entire population, while AZ was distributed most in the age category 45–75 years old and J&J was temporarily indicated only for people >41 years) [6,35]. Additionally, older age, male sex, and presence of certain comorbidities are risk factors for severe COVID-19 [36]. Next, patients that normally resided in nursing homes were distinguished in our models via the variable ‘nursing home residency’ (yes/no). This variable gave insight in the general frailty of an (elderly) patient, which is a risk factor for worse outcomes. In addition, we hypothesized that frail, elderly patients were less frequently admitted to the ICU due to triage as to prevent disproportionate care. Moreover, use of the variable ‘mean ICU occupancy rate’ aimed to adjust for hospital organizational issues that may occur during a surge. The hospital-specific circumstances at the time of a patient’s stay could influence the quality of care they received and thus alter the risk of worse outcomes [37]. The mean ICU occupancy rate (%) was defined as the number of recognized ICU beds per hospital occupied by COVID-19 patients averaged over the patient’s stay in that hospital. Lastly, to account for possible waning of vaccine-induced antibodies, the variable ‘time since vaccination’ (in days) was included in the secondary analysis (see further). It was calculated from the date of administration of the last vaccine dose and the date of hospital admission [12]. The time between receiving a primary vaccination course and infection was related to the exposure variable (being vaccinated) and hypothesized to be associated with the outcome, because waning immunity over time was thought to increase the risk of infection [12] and this could potentially influence disease progression once hospitalized.

### 2.4. Statistical Analyses

Besides descriptive statistics to map the demographics of the main cohort and the DBC subcohort, adjusted odds ratios (ORs) with accompanying 95% confidence intervals (95% CIs) were obtained to estimate the association of the COVID-19 vaccines with severe COVID-19 and in-hospital death. These estimates were obtained through multivariable mixed effects logistic regression analyses after adjusting for relevant covariates (as listed before). In these multilevel models, hospital was considered as the random effect under the assumption of an unstructured correlation matrix. Additionally, only age-adjusted models were reported for the primary analyses.

In the secondary analyses, the effect of the vaccine brand on disease progression to severe COVID-19 and in-hospital death was assessed in the DBC subcohort stratified by age (≤75 years and >75 years). The models were adjusted for age, sex, nursing home residency, comorbidities as listed before, mean ICU occupancy rate, and time since vaccination. Within this smaller DBC subcohort with patients spread over 71 hospitals and after stratification based on age, between-hospital variability was negligible, due to some hospitals only guesting one patient. Because of this and based on Akaike’s information criterion (AIC) and a log likelihood ratio test of a fixed effects logistic regression model and the mixed effects logistic regression model, random effects were removed from the secondary analyses. Logistic regression models with only fixed effects were instead fitted when the between-hospital variance was close to zero. Effect modification by age and the presence or absence of comorbidities on vaccine brand and time since vaccination were explored in the secondary analyses by adding interaction variables and checking their statistical significance, assuming a significance level of 0.05. The conservative Bonferroni method for correcting multiple testing was applied in the secondary models (post hoc pairwise comparisons of the three vaccine brand groups), thus comparing *p* values to an adjusted significance level of 0.0166.

In adjunction to the main analyses, two types of sensitivity analyses were performed. First, a stepwise backward elimination procedure (with significance level of 0.05) was performed to obtain more simplified models. These models were compared to the results of the fully adjusted main models to determine stability of the results and check overfitting. Second, sensitivity analyses were performed, only including data from hospitals with a participation grade of at least 80% during the period of data inclusion. This participation grade was calculated based on the number of patients that were reported by a hospital in the CHS compared to the number of patients obligatorily reported in the exhaustive SCS [25]. The goal of this second type of sensitivity analysis was to test the robustness of the conclusion and shed light on any reporting bias that could have occurred in hospitals with a less than optimal reporting grade.

Assumptions for logistic regression models were checked and no indication of violation of linearity or multicollinearity was found. Results were stable because of the high sample size; fixed explanatory variables had minimal measurement error and observations were independent, while any dependence of observations due to hospital clustering was corrected for by the mixed effects model. As these were complete case analyses, missing data on covariates and loss-to-follow-up of patients due to unavailable outcome data was dealt with by exclusion of these patients. Analyses were performed in R and R Studio (version 4.0.5.). This manuscript was written following the STROBE guidelines.

## 3. Results

### 3.1. Study Population

**Figure 2:**
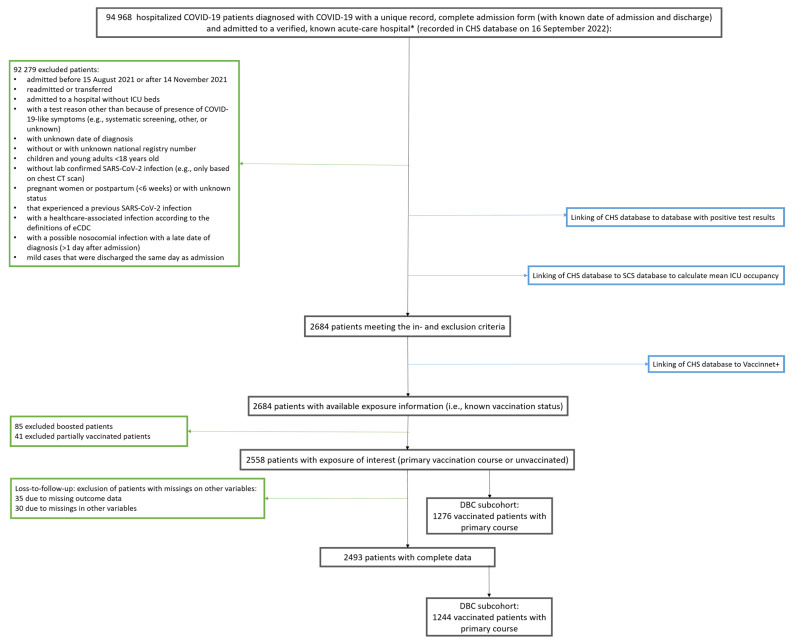
Flowchart for selection of study populations: total cohort and Delta breakthrough cases subcohort. Black boxes show the number of patients throughout the selection process; green boxes show number of excluded patients with reasons for exclusion; blue boxes show linking with databases to obtain information at the indicated exclusion step during the selection process. DBC; Delta breakthrough cases, CHS; clinical hospital surveillance, SCS; surge capacity surveillance, ICU; intensive care unit * no psychiatric or military hospital.

In time period 15 August 2021–14 November 2021, characterized by predominant Delta circulation [27,28,29,30,31], 2684 patients hospitalized in 73 hospitals were captured in the non-exhaustive CHS database who remained eligible after applying in- and exclusion criteria (Figure 2). Of those, 2493 complete cases were identified with available information on the vaccination status of interest (1244 vaccinated with primary vaccination course (49.9%), 1249 unvaccinated (50.1%)) (Table 1). Noteworthily, patients who received a primary vaccination course tended to be older, were more likely to be male, to reside in a nursing home, and to have comorbidities. During the part of the Delta wave included in the study period, patients in our study population on average did not experience a full ICU during their stay (maximum mean ICU occupancy rate was 75%). Of the patients who received a primary vaccination course, 189 were admitted to the ICU, 108 were diagnosed with ARDS and 222 patients died. Severe COVID-19 occurred more often in unvaccinated patients with 298 ICU admissions, 169 ARDS diagnoses and 163 in-hospital deaths. In total, 350 (28.1%) and 386 (30.9%) severe COVID-19 cases were defined in the vaccinated and unvaccinated hospitalized population, respectively. Contrary to the outcome of severe COVID-19, in-hospital death was higher in the vaccinated group than in unvaccinated patients. The cohort after applying the selection criteria, but before excluding losses-to-follow-up and incomplete cases, is described in Table A1. This cohort had similar characteristics as only 2.54% were excluded to obtain the final cohort that was used for analysis.

Of these 2493 patients, 1244 DBCs made up the subcohort. Distribution of the vaccine brands was as following: the majority, 833 patients, received an mRNA vaccine (67%), while 321 patients received AZ (25.8%), and 90 patients received 1 dose of J&J (7.2%) (Table 2). Older people and nursing home residents tended to have received mRNA vaccines with a median age of 77 years old, while J&J was distributed more among younger people with a median age of 70.5 years old. The time since receiving the last vaccine dose varied between the groups that received different brands. Patients that were vaccinated with mRNA vaccines received their last dose a longer time ago (median 168 days and maximum 286 days) than patients that were vaccinated with the adenoviral vector brands, AZ and J&J (median 116 and 115 days, and maximum 171 and 182 days, respectively). Most DBCs had at least one comorbidity, regardless of which vaccine brand they received; however, J&J recipients tended to have fewer comorbidities than recipients of the other brands (most people had multimorbidity of 2, while the median of J&J vaccinated was 1). No large differences in distribution of the vaccine brands across the different type of comorbidities were observed, except for patients with arterial hypertension, who were less represented in the J&J group; only one patient who received a solid organ transplant and no patients suffering from hematological cancer received J&J in our study population. The differences in baseline characteristics between brands were also notable in the number of events in the determinants of interest. Of all severe COVID-19 cases in the DBC subcohort, 244 were mRNA vaccine recipients (29.3% cases of all mRNA vaccine recipients), 76 were AZ recipients (23.7%), and 30 were J&J recipients (33.3%). Of those severe COVID-19 cases, 155, 44, and 23 deaths were observed in the mRNA vaccine (18.6% of all mRNA vaccine recipients), AZ (13.7%), and J&J (25.6%) groups, respectively. Similarly, as for the main cohort, a descriptive table in Table A2 describes the DBC subcohort before exclusion of losses-to-follow-up and incomplete cases. This DBC subcohort and the final subcohort used in the secondary analyses were similar in terms of patient characteristics, as only 2.51% were excluded to obtain the final subcohort.

### 3.2. Effects of Vaccination Status on Disease Progression to Severe COVID-19 and In-Hospital Death

**Figure 3:**
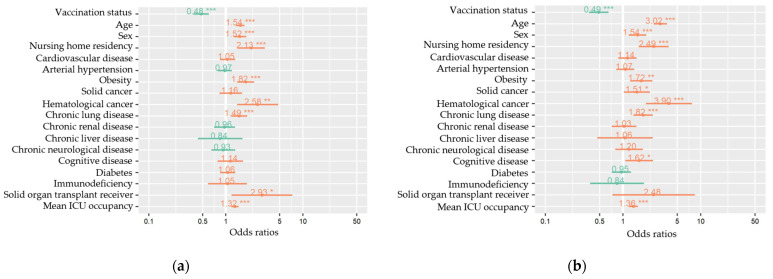
Multivariable mixed-effects models for all measured variables (fixed effects) of severe COVID-19 (**a**) and in-hospital death (**b**) among hospitalized COVID-19 patients, COVID-19 clinical hospital surveillance. The following fixed effects were included in the full models: vaccination status (primary vaccination status compared to unvaccinated), age (years), sex (male compared to female), nursing home residency, cardiovascular disease, arterial hypertension, obesity, solid cancer, hematological cancer, chronic lung disease, chronic renal disease, chronic liver disease, chronic neurological or neuromuscular disease, cognitive disease, diabetes mellitus, immunodeficiency, solid organ transplantation, and mean ICU occupancy rate (%). Hospital was added as a random effect to the model. ICU = intensive care unit. * *p* < 0.05, ** *p* < 0.01, *** *p* < 0.001.

**Figure A1:**
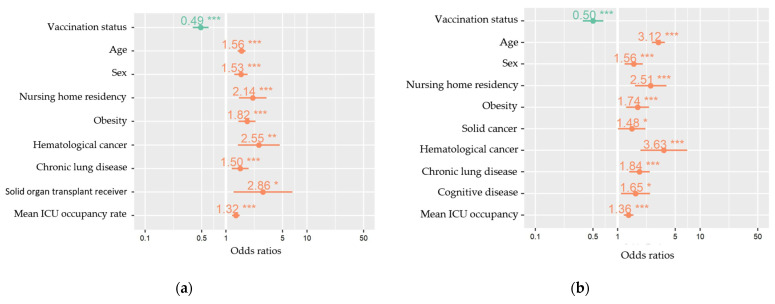
Multivariable mixed-effects models for explanatory variables (fixed effects) of severe COVID-19 (**a**) and in-hospital death (**b**) among hospitalized COVID-19 patients, COVID-19 clinical hospital surveillance. The following fixed effects were retained in the models obtained after stepwise backward elimination of non-significant (*p* < 0.05) variables: vaccination status (primary vaccination course compared to unvaccinated), age (years), sex (male compared to female), nursing home residency, obesity, hematological cancer, chronic lung disease, and mean ICU occupancy rate (%). For the outcome severe COVID-19 (**a**), solid organ transplantation was additionally retained and for in-hospital death (**b**) additionally cognitive disease was retained. Hospital was added as a random effect to the model. ICU = intensive care unit. * *p* < 0.05, ** *p* < 0.01, *** *p* < 0.001.

Within the full cohort of hospitalized COVID-19 patients (N = 2493), mixed effects logistic regression models were composed to estimate the association between vaccination status and severe COVID-19 and in-hospital all-cause death. The odds of developing severe COVID-19 and dying were approximately two times lower for patients vaccinated with a primary course compared to unvaccinated patients after adjustment for age, sex, nursing home residency, comorbidities, mean ICU occupancy rate as fixed effects, and hospital as random effect (odds ratio (OR) 0.48, 95% confidence interval (CI): 0.39–0.61 for severe COVID-19 and OR 0.50, 95% CI: 0.38–0.67 for dying) (Table 3; Figure 3). Sensitivity models after backward selection with estimates for significant variables can be found in Table A3 and Figure A1.

### 3.3. Exploratory Vaccine Brand Comparison among Delta Breakthrough Cases

Among the 607 hospitalized patients > 75 years, the odds of developing severe outcomes when vaccinated with two doses of AZ were about three times lower than when vaccinated with only one dose of J&J (OR 0.37, 95% CI: 0.17–0.80 for severe COVID (Table 4); OR 0.35, 95% CI: 0.16–0.81 for in-hospital death (Table 5), while these odds of severe COVID-19 development were estimated to be about two times lower when vaccinated with mRNA vaccines compared to J&J (OR 0.44, 95% CI: 0.19–1.04 (Table 4)). However, this latter observation was a non-significant trend (*p* = 0.07). In the simplified models in the sensitivity analyses, this suggestive trend of superiority of mRNA vaccines against severe COVID-19 became borderline significant after Bonferroni correction (*p* = 0.017) (Table A4)**.** In the >75 elderly, protection by mRNA vaccines against dying appeared better, with the odds being 2.5 times lower than when vaccinated with J&J (OR 0.42, 95% CI: 0.18–0.97 (Table 5). In the younger patient group (637 patients ≤ 75 years), no significant differences between the effects of the vaccines brands were found in development of severe outcomes (Table 4 and Table 5). For completeness, Table 4 and Table 5 show respective ORs and accompanying 95% CIs for all possible comparisons of brands and the other brands as reference categories. Sensitivity analyses with more simplified models resulted in similar estimates as in the main models with smaller 95% CIs (Table A4 and Table A5) suggesting stability of the results and more precision.

The sensitivity analyses, which included only patients admitted to hospitals that reported consistently, also produced similar estimates for the effects of vaccination status and the vaccine brands in DBCs on critical outcomes. This suggests minimal presence of hospital reporting bias in the main analyses and more robustness in the conclusions that can be drawn from it.

## 4. Discussion

Data linkage within the LINK-VACC framework [26] allowed for explanatory model building to investigate the association between vaccination with a primary vaccination course with mRNA vaccines (MOD and COM), AZ, or J&J and severe COVID-19 and all-cause in-hospital death in patients hospitalized for COVID-19. Vaccination with a primary schedule was associated with lower odds of progression to severe COVID-19 and death after hospitalization for COVID-19. These protective effects were proven before in hospitals in the US [21,22,23], Turkey [24], Slovenia [20], Pakistan [38], Scotland [19], Singapore [10], France [39], and Bahrein [40]. Despite definitions of severe COVID-19 differing across different studies, and some studies including partially vaccinated or boosted patients as ‘fully vaccinated’ or only including mRNA vaccines, ORs estimating the effects against severe COVID-19 ranged from 0.07, 95% CI: 0.01–0.34 [10] to 0.42, 95% CI: 0.2–0.70 [20] for ‘fully vaccinated’ to unvaccinated hospitalized patients [10,20,22,23,24]. For the outcome in-hospital death, ORs of 0.41, 95% CI: 0.19–0.88 and 0.82 (*p* = 0.7) were reported by Tenforde et al. [22] and Tandon et al. [21], respectively. The latter only observed a trend of protective effects by a primary vaccination schedule. However, this estimate was only adjusted for comorbidities and not for age. Our ORs of 0.48, 95% CI: 0.38–0.60 for severe COVID-19 and 0.49, 95% CI: 0.36–0.65 for in-hospital death are in line with these findings in hospitalized patients. 

Following the a priori hypothesis that different vaccine technologies might perform differently in different age categories and after finding a borderline significant result for the interaction between vaccine brand and age for the outcome in-hospital death in preliminary analyses, a stratified analysis was conducted. If there were to be a real effect modification by age, it would be inappropriate to report summarized ORs representing the associations for all age categories together. Two doses of AZ and mRNA vaccines appeared to be less associated with severe COVID-19 and in-hospital death compared to one dose of J&J in patients over 75 years old. In patients of 75 years old or younger this difference between brands was not observed and all brands appeared to protect as effectively against disease progression. A probable explanation is that in the elderly, the phenomenon of immunosenescence and inflammaging is at play and thus J&J, with a one dose vaccine schedule, might be insufficient to mount sufficient immune responses to protect against critical outcomes [41]. These findings support the notion that a second dose would be beneficial for older and vulnerable populations.

Other studies reporting age-stratified models for hospitalized patients included those by Tenforde et al. [22] and Fournier et al. [39]. One the one hand, Tenforde et al. included the mRNA vaccines in their study and reported an adjusted OR of 0.57, 95% CI: 0.27–1.24 for ‘fully vaccinated’ patients <65 years old and an OR of 0.24, 95% CI: 0.11–0.55 for ‘fully vaccinated’ patients >65 years old compared to unvaccinated patients in progression to severe COVID-19 (defined as death or invasive mechanical ventilation) [22]. On the other hand, Fournier et al. included the mRNA vaccines and J&J and chose a cutoff point of 55 years of age to stratify [39]. Similarly though, an adjusted OR of 0.61, 95% CI: 0.04–8.90] was reported for partially or ‘fully vaccinated’ patients <55 years old and an OR of 0.32, 95% CI: 0.16–0.62 was reported for those patients aged ≥55 years compared to unvaccinated patients. Nevertheless, they also selected Alpha-infected patients aside from Delta infections (but adjusted for VOC) to reach a sample size of 11,624. The findings in both studies are in line with our findings. 

When comparing AZ and mRNA vaccines to each other, no differences were detected in their protection against COVID-19 progression. Tenforde et al. [22] did not directly compare the effects of brands, but rather opted for brand-specific ORs compared to unvaccinated patients (0.36, 95% CI: 0.27–0.49 for COM and 0.15, 95% CI: 0.09–0.23 for MOD). Other studies in hospitalized patients reported having insufficient power and group size imbalances that hindered comparison of vaccine brands [20], which also applied to our study; particularly for the MOD group, which is why MOD and COM were combined into 1 group: the mRNA vaccines. A plausible reason for the low number of MOD-vaccinated patients in our study is that COM was more widely distributed in Belgium. We also had a small sample size for the J&J group in the older stratum, as elderly and nursing home residents in Belgium were mostly vaccinated with mRNA vaccines.

### 4.1. Strengths and Limitations

Previously, it was shown how organizational characteristics differences between hospitals (e.g., different clinical profile of admitted patients and different procedures per hospital) can affect patient outcomes [37,42]. A strength of this study was the adjustment for these hospital effects by including hospital as a random effect as well as taking into account the circumstances in the hospital that each patient individually experienced during his/her stay by including the mean ICU occupancy rate. There were no differences between vaccinated and unvaccinated patients based on their mean ICU occupancy rate, which means that vaccination status was not related to the ICU load during a patient’s stay (i.e., patients with a primary vaccination course and unvaccinated patients were both admitted in times with similar ICU occupancy). Mean ICU occupancy rate by itself was a strongly significant factor with a large effect on progression to severe COVID-19 and in-hospital death. This confirms what was seen before in Belgian hospitals [37] and stresses the importance of alleviating pressure on the hospitals and healthcare workers during surges. Another strength is that ‘in-hospital death’ was analyzed separately because ‘severe COVID-19’ included ‘ICU admission’, which can be a subjective clinical outcome as the choice of admission is dependent on the clinician. The decision to transfer patients to the ICU is based on clinical expertise and necessary monitoring conditions, but also depends on available ICU capacity at the moment of uptake.

Time since vaccination was included in the secondary analyses to adjust for waning immunity. This was a strength, since many studies did not adjust for this variable, showing contradicting results to studies that did [12]. Even so, McKeigue et al. note that this variable can still be confounded by other unassessed factors such as seasonality and change in comorbidity profile [19]. Similarly, other factors such as socioeconomic status and ethnicity, that were shown to be associated with vaccine uptake in Belgium [43], could be confounders in the studied association which we did not account for. This potential residual confounding marks a first, important limitation of this study. Residual confounding could also exist from insufficient adjustment for comorbidities (i.e., we did not investigate the nuances in the spectrum of each disease since this information was unavailable, nor multimorbidity or even the possibility of undiagnosed/ incorrectly diagnosed comorbidities that could have caused misclassification). Contrary to residual confounding by socioeconomic status and ethnicity, residual confounding by comorbidities is thought to be limited as adjustment for comorbidities (Table 3) did not attenuate the effect estimate (which would point to overestimation of the protective effects); but even amplified the effect estimate (pointing to potential underestimation due to any residual confounding). Another source of residual confounding could stem from the lack of knowledge of patients’ medication profiles, which affects their immunological state (and consequently their immunological responses to the vaccine and their risk of developing the outcomes).

Other possible biases that the observational, retrospective nature of this design is inherently vulnerable to are selection bias and collider bias. Selection bias could exist as hospital admission bias, where breakthrough cases might be admitted more readily than unvaccinated COVID-19 cases or similarly if vaccinated patients were more conscious of their health, resulting in increased healthcare seeking behavior. If this happened, it could have led to an underestimation of the protective effects of the vaccines. Other studies checked the influence of this hospital admission bias with sensitivity analyses restricting on patients with hypoxemia as a more objective biomarker [20,23], but to us this information was unavailable. Changes in criteria for hospitalization during the inclusion period (e.g., due to the increased availability of therapeutic options) could be additional causes of selection bias [44]. Nevertheless, mean ICU occupancy rate per patient reflected relatively stable circumstances in hospitals and the provided quality of care, which could be an argument against too many changes in the threshold for hospitalization due to hospital oversaturation. Patients with complex, unclear transfer history were excluded from our study and this could result in exclusion bias. This was expected to have a low influence on the results as only a low number of this patient type was excluded. In contrast, Griffith et al. propose the high risk of collider bias that exists in COVID-19 studies that use a sample of hospitalized patients [45]. All variables associated with hospitalization could result in spurious associations due to selecting based on these variables. In other words, it could be that, e.g., comorbidities that are strong risk factors for hospitalization are less relevant for disease worsening once hospitalized, potentially creating colliders. This may have been partially solved by adjustment for comorbidities in the multivariable analysis, but residual collider effects cannot be ruled out and selecting on hospitalization may thus still pose a threat for the internal and external validity of our study results.

Other considerations are the following. No sequencing data were available to confirm if all SARS-CoV-2 infections were caused by the Delta variant, however baseline surveillance in Belgium reported 99–100% of the infections during the time of inclusion to be of Delta origin [27,28,29,30,31]. The outcome ‘in-hospital death’ was defined as all-cause mortality, which is not the same as COVID-19-confirmed mortality. Possible misclassification of cases could exist due to this lack of available data on causes of death; however, all-cause mortality remains an important, objective, hard outcome. Lastly, the studied outcomes are important life-threatening complications, but these are not substitutes for biological markers of the pathophysiology of COVID-19, nor the characterization of the immunological response to the vaccines. Even when using a proxy such as time since vaccination to gain understanding on contracting humoral immunity, we did not have data on the actual immune responses in patients and particularly not on the immune status before hospitalization. Additionally, Tenforde et al. make a good point that these types of epidemiological studies do not accurately characterize disease stages from COVID-19 to severe COVID-19 [23].

### 4.2. Generalizability

The conclusions drawn from this study are only valid for hospitalized patients in Belgium. They should not be applied to the general population, outpatients such as nursing home residents that were not hospitalized (due to triage/resource allocation or prioritization of other patients), or moderate and mild COVID-19 cases, nor to hospitals in other countries (due to differences in healthcare systems, hospital characteristics, and admission criteria). Despite these constraints on generalizability, a strength of this study was its multicenter nature, that allowed for increased external validity of the results to patients in all acute-care hospitals across Belgium. 

The estimates for the odds ratios (ORs) reflected the effect of a full vaccination schedule compared to no vaccination on disease progression starting from the time point of hospitalization. It is unknown from this study what the risk factors for progression are from earlier disease stages, nor for progression to other outcomes. From this study, no conclusions can be made on the effects of booster doses of the brands. On the other hand, since the majority of the Belgian population has received one or more vaccine doses and/or has come into contact with SARS-CoV-2, it is becoming increasingly difficult to compare the effects of boosters with unvaccinated or completely naïve subgroups, which this study was still able to do. This study focused on COVID-19 as a result of a Delta infection, so estimates for the effects of the vaccines are different for protection against other VOCs (as also observed by reported VE studies). Currently, Omicron and sublineages of this VOC are circulating, having completely outplaced Delta. This suggests decreased relevance of these results in current times; however, knowledge on the vaccines and how they act against different variants prepares us for the potential emergence of other VOCs and provides reference information when VOCs and different waves are compared in other studies.

Finally, the aim of this study was to explain the protective effect of COVID-19 vaccines and to explore potential differences between brands on the outcomes in this specific patient population with appropriate adjustment for relevant confounders. Further studies using directed acyclic graphs or causal mediation analysis would be a logical step in the follow-up of these results.

## 5. Conclusions

Full vaccination with mRNA vaccines (mRNA-1273 and BNT162b2), ChAdOx1 (AZ), and Ad26.COV2.S (J&J) protected against disease worsening to severe COVID-19 and in-hospital death in hospitalized COVID-19 patients. Exploratory analyses suggested that two doses of AZ and mRNA vaccines might be superior to one dose of J&J in elderly hospitalized patients and that this protective effect might be related to age, while two-dose vaccine brands AZ and the mRNA vaccines did not seem to differ in their protection against critical outcomes after hospitalization.

## Figures and Tables

**Table 1:** Description study population (complete cases).

Characteristics	Total Cohort *n* (%) 2493	Primary Vaccination Course *n* (%) 1244 (49.9)	Unvaccinated *n* (%) 1249 (50.1)
Baseline characteristics			
Age in years, median (IQR) [range]	67 (52–67) [18–112]	75 (65–75) [18–100]	56 (43–56) [18–112]
Sex			
Women	1043 (41.8)	486 (39.1)	557 (44.6)
Men	1450 (58.2)	758 (60.9)	692 (55.4)
Nursing home residency			
Nursing home resident	131 (5.3)	109 (8.8)	22 (1.8)
Living with a level of independence	2362 (94.7)	1135 (91.2)	1227 (98.2)
Presence of comorbidities			
No comorbidities	611 (24.5)	147 (11.8)	464 (37.1)
Comorbidities	1882 (75.5)	1197 (88.2)	785 (62.9)
Cardiovascular disease	700 (28.1)	508 (40.8)	192 (15.4)
Arterial hypertension	903 (36.2)	543 (43.6)	360 (28.8)
Diabetes	556 (22.3)	327 (26.3)	229 (18.3)
Chronic renal disease	270 (10.8)	194 (15.6)	76 (6.1)
Chronic liver disease	51 (2)	30 (2.4)	21 (1.7)
Chronic neurologic or neuromuscular disease	191 (7.7)	134 (10.8)	57 (4.6)
Cognitive impairment	157 (6.3)	116 (9.3)	41 (3.3)
Immunocompromised disease	80 (3.2)	59 (4.7)	21 (1.7)
Chronic lung disease	439 (17.6)	313 (25.2)	126 (10.1)
Solid cancer	201 (8.1)	144 (11.6)	57 (4.6)
Hematological cancer	52 (2.1)	38 (3.1)	14 (1.1)
Solid organ transplantation	26 (1)	23 (1.8)	3 (0.2)
Obesity	406 (16.3)	166 (13.3)	240 (19.2)
Number of comorbidities, median [range]	1 [0–8]	2 [0–7]	1 [0–8]
Determinants			
Mean ICU occupancy rate in %, median (IQR) [range]	18.7 (10.8–18.7) [0–75]	18.7 (10.9–18.7) [0–69.2]	18.8 (10.7–18.8) [0–75]
Length of stay in days, median (IQR) [range]	7 (4–7) [1–193]	8 (5–8) [1–147]	7 (4–7) [1–193]
ICU admission	487 (19.5)	189 (15.2)	298 (23.9)
ARDS	277 (11.1)	108 (8.7)	169 (13.5)
In-hospital death	385 (15.4)	222 (17.8)	163 (13.1)
Severe COVID-19	736 (29.5)	350 (28.1)	386 (30.9)

**Table 2:** Description Delta breakthrough cases subcohort (complete cases).

Characteristics	mRNA *n* (%) 833 (67)	AZ *n* (%) 321 (25.8)	J&J *n* (%) 90 (7.2)
Baseline characteristics			
Age in years, median (IQR) [range]	77 (69–77) [18–98]	73 (63–73) [22–100]	70.5 (54–70.5) [29–99]
Sex			
Women	327 (39.2)	127 (39.1)	32 (35.6)
Men	506 (60.7)	194 (60.4)	58 (64.4)
Nursing home residency			
Nursing home resident	97 (11.6)	9 (2.8)	3 (3.3)
Living with a level of independence	736 (88.4)	312 (97.2)	87 (96.7)
Presence of comorbidities			
No comorbidities	83 (10)	40 (12.5)	24 (26.7)
Comorbidities	750 (90)	281 (87.5)	66 (73.3)
Cardiovascular disease	343 (41.2)	134 (41.7)	31 (34.4)
Arterial hypertension	381 (45.7)	134 (41.7)	28 (31.1)
Diabetes	216 (25.9)	91 (28.3)	20 (22.2)
Chronic renal disease	135 (16.2)	50 (15.6)	9 (10)
Chronic liver disease	19 (2.3)	8 (2.5)	3 (3.3)
Chronic neurologic or neuromuscular disease	96 (11.5)	32 (10)	6 (6.7)
Cognitive impairment	82 (9.8)	25 (7.8)	9 (10)
Immunocompromised disease	40 (4.8)	18 (5.6)	1 (1.1)
Chronic lung disease	213 (25.6)	78 (24.3)	22 (24.4)
Solid cancer	94 (11.3)	41 (12.8)	9 (10)
Hematological cancer	32 (3.8)	6 (1.9)	0
Solid organ transplantation	10 (1.2)	12 (3.7)	1 (1.1)
Obesity	114 (13.7)	41 (12.8)	11 (12.2)
Number of comorbidities, median [range]	2 [0–7]	2 [0–7]	1 [0–6]
Time since vaccination in days, median (IQR) [range]	168 (144–168) [14–286]	116 (95–116) [24–171]	115 (84–115) [14–182]
Determinants			
Mean ICU occupancy rate in %, median (IQR) [range]	18.6 (11.3–18.6) [0–69.1]	18.3 (10.5–18.3) [0–69.2]	19.2 (10–19.2) [0–63]
Length of stay in days, median (IQR) [range]	8 (5–8) [1–141]	7 (4–7) [1–147]	8 (4–8) [1–95]
ICU admission	134 (16.1)	45 (14)	10 (11.1)
ARDS	76 (9.1)	26 (8.1)	6 (6.7)
Death	155 (18.6)	44 (13.7)	23 (25.6)
Severe COVID-19	244 (29.3)	76 (23.7)	30 (33.3)

**Table 3:** Primary results.

Odds Ratios and 95% Confidence Intervals for Severe COVID-19 and Death				
Model	Severe COVID-19		Death	
	OR	95% CI	OR	95% CI
Only age-adjusted mixed effects logistic regression	0.5973 ^A^	0.4842; 0.7368	0.6678 ^B^	0.5082; 0.8776
Full mixed effects logistic regression	0.4793 ^C^	0.3817; 0.6018	0.4876 ^D^	0.3646; 0.6520

^A,B^ Adjusted for age and hospital as random effect. ^C,D^ Adjusted for age, sex, nursing home residency, comorbidities: cardiovascular disease, arterial hypertension, diabetes, chronic renal disease, chronic liver disease, chronic neurologic or neuromuscular disease, cognitive impairment, immunocompromised disease (including HIV and chronic corticosteroid use), chronic lung disease, solid cancer, hematological cancer, solid organ transplantation and obesity, mean ICU occupancy rate, and hospital as random effect.

**Table 4:** Secondary results stratified on age for outcome of severe COVID-19.

Odds Ratios and 95% Confidence Intervals for Severe COVID-19			
Age ≤ 75 y Old ^F^			
Ref category →	J&J	AZ	mRNA
J&J		1.59 (0.72; 3.50)	1.05 (0.48; 2.28)
AZ	0.63 (0.29; 1.39)		0.66 (0.39; 1.11)
mRNA	0.95 (0.44; 2.07)	1.51 (0.90; 2.53)	
Age > 75 y old ^G^			
J&J		**2.61 (1.17; 5.78)**	2.08 (0.93; 4.68)
AZ	**0.38 (0.17; 0.85)**		0.80 (0.45; 1.4)
mRNA	0.44 (0.19; 1.04)	1.25 (0.70; 2.23)	

^F^ Adjusted for age, sex, nursing home residency, presence or absence of comorbidities, comorbidities separately: cardiovascular disease, arterial hypertension, diabetes, chronic renal disease, chronic liver disease, chronic neurologic or neuromuscular disease, cognitive impairment, immunocompromised disease (including HIV and chronic corticosteroid use), chronic lung disease, solid cancer, hematological cancer, solid organ transplantation and obesity, mean ICU occupancy rate, time since vaccination, and age x time since vaccination (interaction). ^G^ Adjusted for age, sex, nursing home residency, presence or absence of comorbidities, comorbidities separately: cardiovascular disease, arterial hypertension, diabetes, chronic renal disease, chronic liver disease, chronic neurologic or neuromuscular disease, cognitive impairment, immunocompromised disease (including HIV and chronic corticosteroid use), chronic lung disease, solid cancer, hematological cancer, solid organ transplantation and obesity, mean ICU occupancy rate, and time since vaccination. → shows the reference category that was used in the different models. Bold numbers signal statistically significant observations (*p* < 0.05).

**Table 5:** Secondary results stratified on age for outcome in-hospital death.

Odds Ratios and 95% Confidence Intervals for In-hospital Death			
Age ≤ 75 y Old ^H^			
Ref category →	J&J	AZ	mRNA
J&J		2.37 (0.78; 7.20)	1.37 (0.46; 4.13)
AZ	0.42 (0.14; 1.28)		0.58 (0.27; 1.24)
mRNA	0.73 (0.24; 2.19)	1.73 (0.81; 3.70)	
Age > 75 y old ^I^			
J&J		**2.82 (1.24; 6.42)**	**2.28 (1.03; 5.50)**
AZ	**0.35 (0.16; 0.81)**		0.84 (0.45; 1.58)
mRNA	**0.42 (0.18; 0.97)**	1.19 (0.63; 2.21)	

^H^ Adjusted for age, sex, nursing home residency, presence or absence of comorbidities, comorbidities separately: cardiovascular disease, arterial hypertension, diabetes, chronic renal disease, chronic liver disease, chronic neurologic or neuromuscular disease, cognitive impairment, immunocompromised disease (including HIV and chronic corticosteroid use), chronic lung disease, solid cancer, hematological cancer, solid organ transplantation and obesity, mean ICU occupancy rate, and time since vaccination. ^I^ Adjusted for age, sex, nursing home residency, presence or absence of comorbidities, comorbidities separately: cardiovascular disease, arterial hypertension, diabetes, chronic renal disease, chronic liver disease, chronic neurologic or neuromuscular disease, cognitive impairment, immunocompromised disease (including HIV and chronic corticosteroid use), chronic lung disease, solid cancer, hematological cancer, solid organ transplantation and obesity, mean ICU occupancy rate, and time since vaccination. → shows the reference category that was used in the different models. Bold numbers signal statistically significant observations (*p* < 0.05).

## Data Availability

The individual level datasets generated or analyzed during the current study do not fulfill the requirements for open data access. The data is too dense and comprehensive to preserve patient privacy. The data of the individual data sources (Clinical Hospital Survey, Vaccinnet+, COVID-19 TestResult Database) within the LINK-VACC project are kept in the pseudonymized environment of healthdata.be and a link between the individual data in each of them takes place thanks to the use of a pseudonymized national reference number managed by healthdata.be under a “project mandate”. A “project mandate” consists of a group of individuals, a group of variables, and a time period. Access rights to the pseudonymized data in the healthdata.be data warehouse are granted ad nominatum for the scientists involved in the surveillance activities at Sciensano. External investigators with a request for selected data should fill in the data request form (https://epistat.sciensano.be/datarequest/, accessed on 11 November 2022). Depending on the type of desired data (anonymous or pseudonymized), the provision of data will have to be assessed by the Belgian Information Security Committee Social Security & Health based on legal and ethical regulations, and is outlined in a data transfer agreement with the data owner (Sciensano).

## Appendix A

**Table A1:** Description study population before exclusion of losses-to-follow-up.

Characteristics	Total Cohort *n* (%) 2558	Primary Vaccination Course *n* (%) 1276 (49.9)	Unvaccinated *n* (%) 1282 (50.1)
Baseline characteristics			
Age in years, median (IQR) [range]	67 (53.5–80.5) [18–112]	75 (66–84) [18–100]	56 (43.1–68.9) [18–112]
Sex			
Women	1065 (41.6)	498 (39)	567 (44.2)
Men	1493 (58.4)	778 (61)	715 (55.8)
Nursing home residency			
Nursing home resident	136 (5.3)	113 (8.9)	23 (1.8)
Living with a level of independence	2394 (93.6)	1145 (89.7)	1249 (97.4)
Presence of comorbidities			
No comorbidities	627 (24.5)	150 (11.8)	477 (37.2)
Comorbidities	1931 (75.5)	1126 (88.2)	805 (62.8)
Cardiovascular disease	720 (28.1)	519 (40.7)	201 (15.7)
Arterial hypertension	927 (36.2)	560 (43.9)	367 (28.6)
Diabetes	575 (22.5)	341 (26.7)	234 (18.3)
Chronic renal disease	277 (10.8)	200 (15.7)	77 (6)
Chronic liver disease	57 (2.2)	32 (2.5)	25 (2)
Chronic neurologic or neuromuscular disease	197 (7.7)	139 (10.9)	58 (4.5)
Cognitive impairment	160 (6.3)	119 (9.3)	41 (3.2)
Immunocompromised disease	82 (3.2)	61 (4.8)	21 (1.6)
Chronic lung disease	454 (17.7)	324 (25.4)	130 (10.1)
Solid cancer	204 (8.0)	147 (11.5)	57 (4.4)
Hematological cancer	53 (2.1)	39 (3.1)	14 (1.1)
Solid organ transplantation	26 (1)	23 (1.8)	3 (0.2)
Obesity	414 (16.2)	171 (13.4)	243 (19)
Number of comorbidities, median [range]	1 [0–8]	2 [0–8]	1 [0–8]
Determinants			
Mean ICU occupancy rate in %, median (IQR) [range]	18.5 (10.2–26.8) [0–75]	18.5 (10.7–26.4) [0–69.2]	18.5 (9.8–27) [0–75]
Length of stay in days, median (IQR) [range]	7 (2.5–11.5) [1–365]	8 (3.5–12.5) [1–365]	7 (3–11) [1–193]
ICU admission	500 (19.5)	194 (15.2)	306 (23.9)
ARDS	297 (11.6)	113 (8.9)	184 (14.4)
In-hospital death	400 (15.6)	228 (17.9)	172 (13.4)
Severe COVID-19	767 (30)	360 (28.2)	407 (31.7)

**Table A2:** Description Delta breakthrough cases subcohort before exclusion of losses-to-follow-up.

Characteristics	mRNA *n* (%) 854 (66.9)	AZ *n* (%) 331 (25.9)	J&J *n* (%) 91 (7.1)
Baseline characteristics			
Age in years, median (IQR) [range]	67 (53.5–80.5) [18–112]	75 (66–84) [18–100]	56 (43.1–68.9) [18–112]
Sex			
Women	1065 (41.6)	498 (39)	567 (44.2)
Men	1493 (58.4)	778 (61)	715 (55.8)
Nursing home residency			
Nursing home resident	136 (5.3)	113 (8.9)	23 (1.8)
Living with a level of independence	2394 (93.6)	1145 (89.7)	1249 (97.4)
Presence of comorbidities			
No comorbidities	627 (24.5)	150 (11.8)	477 (37.2)
Comorbidities	1931 (75.5)	1126 (88.2)	805 (62.8)
Cardiovascular disease	720 (28.1)	519 (40.7)	201 (15.7)
Arterial hypertension	927 (36.2)	560 (43.9)	367 (28.6)
Diabetes	575 (22.5)	341 (26.7)	234 (18.3)
Chronic renal disease	277 (10.8)	200 (15.7)	77 (6)
Chronic liver disease	57 (2.2)	32 (2.5)	25 (2)
Chronic neurologic or neuromuscular disease	197 (7.7)	139 (10.9)	58 (4.5)
Cognitive impairment	160 (6.3)	119 (9.3)	41 (3.2)
Immunocompromised disease	82 (3.2)	61 (4.8)	21 (1.6)
Chronic lung disease	454 (17.7)	324 (25.4)	130 (10.1)
Solid cancer	204 (8.0)	147 (11.5)	57 (4.4)
Hematological cancer	53 (2.1)	39 (3.1)	14 (1.1)
Solid organ transplantation	26 (1)	23 (1.8)	3 (0.2)
Obesity	414 (16.2)	171 (13.4)	243 (19)
Number of comorbidities, median [range]	1 [0–8]	2 [0–8]	1 [0–8]
Time since vaccination in days, median (IQR) [range]	67 (53.5–80.5) [18–112]	75 (66–84) [18–100]	56 (43.1–68.9) [18–112]
Determinants			
Mean ICU occupancy rate in %, median (IQR) [range]	18.5 (10.2–26.8) [0–75]	18.5 (10.7–26.4) [0–69.2]	18.5 (9.8–27) [0–75]
Length of stay in days, median (IQR) [range]	7 (2.5–11.5) [1–365]	8 (3.5–12.5) [1–365]	7 (3–11) [1–193]
ICU admission	500 (19.5)	194 (15.2)	306 (23.9)
ARDS	297 (11.6)	113 (8.9)	184 (14.4)
Death	400 (15.6)	228 (17.9)	172 (13.4)
Severe COVID-19	767 (30)	360 (28.2)	407 (31.7)

## Appendix B

**Table A3:** Simplified model after stepwise backward selection.

Odds Ratios and 95% Confidence Intervals for Severe COVID-19 and Death				
Model	Severe COVID-19		Death	
	OR	95% CI	OR	95% CI
Mixed effects logistic regression (only significant variables)	0.4852 ^J^	0.3874; 0.6077	0.5026 ^K^	0.3769; 0.6703

^J^ Adjusted for age, sex, nursing home residency, comorbidities: hematological cancer, solid organ transplantation, obesity, chronic lung disease, mean ICU occupancy rate, and hospital as random effect. ^K^ Adjusted for age, sex, nursing home residency, comorbidities: hematological cancer, solid cancer, chronic lung disease, obesity and cognitive issues, mean ICU occupancy rate, and hospital as random effect.

## Appendix C

**Table A4:** Secondary results stratified on age for the outcome severe COVID-19.

Odds Ratios and 95% Confidence Intervals for Severe COVID-19			
Age ≤ 75 y old ^L^			
Ref category →	J&J	AZ	mRNA
J&J		1.55 (0.72; 3.37)	0.94 (0.44; 2.00)
AZ	0.64 (0.30; 1.40)		0.61 (0.37; 1.01)
mRNA	1.06 (0.50; 2.25)	1.65 (0.99; 2.73)	
Age > 75 y old ^M^			
J&J		**2.71 (1.24; 5.90)**	**2.34 (1.16; 4.72)**
AZ	**0.37 (0.17; 0.80)**		0.86 (0.55; 1.37)
mRNA	**0.43 (0.21; 0.86)**	1.16 (0.73; 1.83)	

^L^ Adjusted for age, obesity, chronic lung disease, cognitive impairment, solid organ transplantation, mean ICU occupancy rate, time since vaccination, and age x time since vaccination (interaction). ^M^ Adjusted for sex, nursing home residency, and hematological cancer. Bold numbers signal statistically significant observations (*p* < 0.05).

**Table A5:** Secondary results stratified on age for outcome in-hospital death.

Odds Ratios and 95% Confidence Intervals for In-hospital Death			
Age ≤ 75 y Old ^N^			
Ref category →	J&J	AZ	mRNA
J&J		2.00 (0.69; 5.82)	1.50 (0.56; 4.00)
AZ	0.50 (0.17; 1.45)		0.75 (0.40; 1.40)
mRNA	0.67 (0.25; 1.78)	1.33 (0.72; 2.49)	
Age > 75 y old ^O^			
J&J		**3.13 (1.40; 7.01)**	**3.05 (1.48; 6.26)**
AZ	**0.32 (0.14; 0.72)**		0.97 (0.59; 1.60)
mRNA	**0.32 (0.16; 0.67)**	1.03 (0.62; 1.69)	

^N^ Adjusted for age, nursing home residency, chronic lung disease, chronic renal disease, and mean ICU occupancy rate. ^O^ Adjusted for sex, nursing home residency, chronic lung disease, chronic liver disease, and hematological cancer. Bold numbers signal statistically significant observations (*p* < 0.05).
